# Mitochondrial nucleic acids in innate immunity and beyond

**DOI:** 10.1038/s12276-023-01121-x

**Published:** 2023-12-01

**Authors:** Jimin Yoon, Sujin Kim, Mihye Lee, Yoosik Kim

**Affiliations:** 1 Department of Chemical and Biomolecular Engineering, Korea Advanced Institute of Science and Technology (KAIST), Daejeon, 34141 Republic of Korea; 2 Soonchunhyang Institute of Medi-bio Science, Soonchunhyang University, Cheonan, 31151 Republic of Korea; 3 Department of Integrated Biomedical Science, Soonchunhyang University, Cheonan, 31151 Republic of Korea; 4 Graduate School of Engineering Biology, KAIST, Daejeon, 34141 Republic of Korea; 5 KAIST Institute for BioCentury (KIB), KAIST, Daejeon, 34141 Republic of Korea; 6 KAIST Institute for Health Science and Technology (KIHST), KAIST, Daejeon, 34141 Republic of Korea; 7 BioProcess Engineering Research Center and BioInformatics Research Center, KAIST, Daejeon, 34141 Republic of Korea

**Keywords:** Long non-coding RNAs, RNA metabolism, Pattern recognition receptors

## Abstract

Mitochondria participate in a wide range of cellular processes. One essential function of mitochondria is to be a platform for antiviral signaling proteins during the innate immune response to viral infection. Recently, studies have revealed that mitochondrion-derived DNAs and RNAs are recognized as non-self molecules and act as immunogenic ligands. More importantly, the cytosolic release of these mitochondrial nucleic acids (mt-NAs) is closely associated with the pathogenesis of human diseases accompanying aberrant immune activation. The release of mitochondrial DNAs (mtDNAs) via BAX/BAK activation and/or VDAC1 oligomerization activates the innate immune response and inflammasome assembly. In addition, mitochondrial double-stranded RNAs (mt-dsRNAs) are sensed by pattern recognition receptors in the cytosol to induce type I interferon expression and initiate apoptotic programs. Notably, these cytosolic mt-NAs also mediate adipocyte differentiation and contribute to mitogenesis and mitochondrial thermogenesis. In this review, we summarize recent studies of innate immune signaling pathways regulated by mt-NAs, human diseases associated with mt-NAs, and the emerging physiological roles of mt-NAs.

## Mitochondria-derived DNAs and RNAs: the hidden triggers of innate immune response

Mitochondrial nucleic acids (mt-NAs) play a crucial role in activating various innate immune signaling pathways and are associated with numerous human diseases. Downregulation of mt-NAs or their downstream receptors often alleviates interferon signature. Recent studies reveal that mt-NAs can also serve as key mediators of signaling between mitochondria and the nucleus, thereby contributing to cell physiology. In particular, studies have shown that mt-NAs can stimulate beige adipocyte development in young mice, which has potential therapeutic implications for obesity and insulin resistance. Understanding the multifaceted roles of mt-NAs will provide potential therapeutic strategies for mt-NA-mediated immune signaling regulation in pathophysiology.

This summary was initially drafted using artificial intelligence, then revised and fact-checked by the author.

## Introduction

Mitochondria are semi-independent double membrane-bound organelles with their own DNA genome. The mitochondrial genome is characterized by a circular, double-stranded structure comprised of 37 genes, 13 of which encode subunits of oxidative phosphorylation (OXPHOS) complex, making mitochondria essential energy-generation centers of the cell^1^. In addition to ATP production, mitochondria are responsible for carrying out a wide range of cellular processes. The mitochondrial matrix stores calcium ions, which promote ATP synthesis under physiological conditions and increase mitochondrial permeability to facilitate necrosis under stress conditions^2^. Moreover, mitochondria generate reactive oxygen species (ROS) that can damage DNA in the nucleus and promote cellular senescence^3^. Last but not least, mitochondria are essential in mediating innate immune responses, serving as central hubs of cellular defense systems^4^.

Traditionally, the immune function of mitochondria in response to viral infection has been focused on mitochondrial antiviral signaling proteins (MAVS) located on the mitochondrial outer membrane (MOM)^5^. The caspase recruitment domain of MAVS interacts with oligomerized retinoic acid-inducible gene-I (RIG-I)-like receptors (RLRs), such as RIG-I and melanoma differentiation-associated protein 5 (MDA5), that recognize double-stranded RNAs (dsRNAs) in the cytosol^6^. This interaction leads to the activation of nuclear factor-κB (NF-κB) and interferon regulatory factor 3/7 (IRF3/7), resulting in the transcription of proinflammatory cytokines and type I interferons (IFN-I)^7^. Therefore, by hosting MAVS on the MOM, mitochondria are the subcellular organelles in which the innate immune response by RLRs is initiated.

Recently, innate immunity to mitochondrial nucleic acids (mt-NAs) has been receiving increasing attention. The human immune system evades and protects cells from aberrant immune activation to endogenous (self) NAs through epigenetic modifications, such as DNA methylation. Indeed, the accumulation of hypomethylated DNAs in the cytosol due to mitotic defects and genomic instability trigger cyclic GMP-AMP synthase (cGAS)-dependent innate immune activation, resulting in the acquisition of autoinflammatory phenotypes^8^. However, mt-NAs do not undergo modifications as they are sequestered inside mitochondria away from cytosolic sensors; therefore, they do not evade immune system surveillance. As a result, these mt-NAs can be recognized and trigger an aberrant immune response when present in the cytosol. In particular, the unique circular structure of mitochondrial DNA (mtDNA) makes them sentinels of the innate immune system that recognize non-self molecules^9^. Moreover, the A-form helical structure of mitochondrial double-stranded RNAs (mt-dsRNAs), generated during bidirectional transcription of the circular mitochondrial genome, is recognized by dsRNA sensors and activates the IFN-I pathway. A study by Young and Attardi^10^ provided biochemical evidence to support the existence of long dsRNAs in human mitochondria. Recently, by utilizing J2 monoclonal antibodies that specifically recognize dsRNAs, Dhir et al. and Kim et al. independently confirmed the presence of cytosolic mt-dsRNAs^11,12^. More importantly, these cytosolic mt-dsRNAs are considered foreign NAs by our immune surveillance system, and therefore, mt-dsRNAs activate innate immune signaling. Interestingly, recent studies have reported the possible physiological function of cytosolic mt-NAs during beige adipocyte development, suggesting that the mt-NAs may have a function beyond triggering aberrant immune activation and potentially transmit signals from mitochondria to the nucleus^13^. Overall, this review summarizes the role of mt-NAs as immunogenic ligands, particularly in the development of human diseases that accompany aberrant immune activation, and provides perspectives on the emerging physiological function of mt-NAs. Understanding the multifaceted roles of mt-NAs may lead to a potential therapeutic strategy for mt-NA-mediated immune signaling regulation in pathophysiological contexts.

### Mechanisms of the cytosolic release of mt-NAs

The cytosolic release of mt-NAs contributes to the activation of innate immune response systems, which in turn often leads to pathological consequences. It is important to understand how mt-NAs escape from mitochondria and are recognized by cytosolic pattern recognition receptors (PRRs). Several reports have indicated that the loss of mitochondrial integrity and compartmentalization due to mitochondrial stress could result in the cytosolic release of mt-NAs^14^. These mitochondrial stressors include the excessive accumulation of ROS due to an imbalance in ROS generation and antioxidant defense mechanism pathways and disruption to the mitochondrial network, resulting in mitochondria fragmentation characterized by reduced mitochondrial volume and increased mitochondrial sphericity. For example, during measles virus infection, the viral V protein sequesters the mitochondrial enzyme aminolevulinate synthase in the cytosol to disrupt the mitochondrial metabolism, which subsequently facilitates the fragmentation and degradation of the mitochondrial network, leading to the mtDNA release into the cytosol^15^. Similarly, herpes simplex virus type 1 infection also triggers the cytosolic efflux of mtDNAs by facilitating mitochondrial fragmentation^16^.

**Fig. 1:**
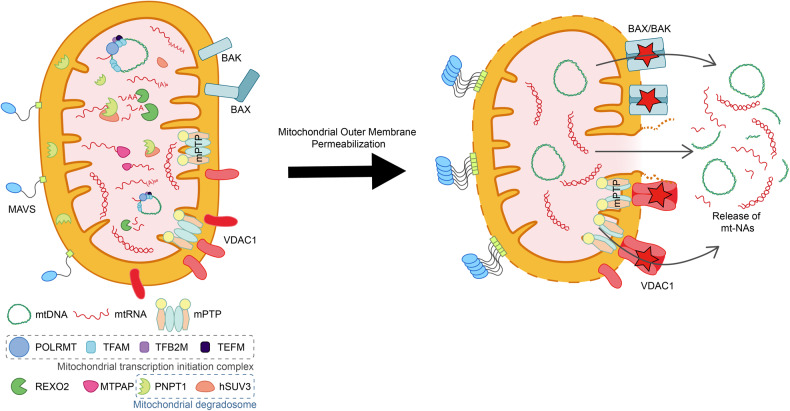
Efflux of mt-NAs via MOMP. Under cellular stress, BAX and BAK are activated, leading to MOMP. MIM permeabilization and efflux of mt-NAs involve herniation through BAX/BAK macropores. The release of mt-NAs is also mediated by mPTP and VDAC1 oligomerization.

The release of mtDNAs is mediated by two types of MOM pores (Fig. 1). First, voltage-dependent anion channel 1 (VDAC1) forms oligomers and creates large pores on the MOM to allow the transportation of metabolites and mtDNAs across the mitochondrial membrane^17^. In this context, mtDNAs must pass through the poorly defined mitochondrial permeability transition pores (mPTPs) located at the mitochondrial inner membrane (MIM)^18^. mPTPs are transmembrane proteins in the MIM and responsible for the permeabilization of the MOM (MOMP)^19^. Normally, mPTPs are closed, but they can open under specific conditions such as during a response to increased mitochondrial Ca^2+^ concentration^20^. VDAC1 participates in the formation of mPTP complexes and triggers the opening of mPTPs under oxidative stress conditions, thereby acting as an ROS sensor^17^. Notably, the transcriptional activity of VDAC1 can be stimulated under starvation and oxidative stress conditions, suggesting that the release of mtDNAs might be a programmed stress response^21^. Moreover, during their release, mtDNAs interact with multiple VDAC1 molecules through the positively charged residues in the N-terminal domain and act as scaffolds to stabilize the VDAC1 oligomers, thereby promoting their own cytosolic efflux^17^. This positive feedback action is promoted under inflammatory conditions to enhance the mtDNA-mediated immune response. Indeed, inhibiting VDAC1 oligomerization alleviates inflammatory response triggered by cytosolic mtDNAs^17^.

In addition to VDAC1-mediated efflux, mtDNAs can be released to the cytosol via pores created by B-cell leukemia/lymphoma 2 (BCL2)-associated X (BAX) and BCL2 antagonist/killer (BAK) proteins. In response to several cellular stressors, free cytosolic BAX proteins are recruited to the MOM and oligomerize with BAK via the exposed BH3 domain in BAK^22^. As the BAX/BAK oligomers assemble, macropores form, and the MIM herniates and opens into the cytosol, secreting mtDNAs, mitochondrial RNAs (mtRNAs), and other mitochondrial components^23^. The main difference between VDAC1 and BAX/BAK pores is that VDAC1 oligomers can form both in healthy and in stressed cells and require the opening of the mPTP opening to permeabilize the MIM. In contrast, BAX/BAK macropores form only under severe stress conditions, and their formation often leads to apoptosis^23,24^.

A recent study demonstrated VDAC-mediated cytosolic release of mtDNAs under pyrimidine-deficient conditions^25^. In this context, mtDNA release was determined by the expression of mitochondrial protease YME1-like 1 ATPase (YME1L), which preserves cytosolic nucleotide pools by synthesizing pyrimidine from glutamine and limiting the accumulation of the mitochondrial pyrimidine transporter SLC25A33^25^. The decreased expression of YME1L resulted in the fragmentation of the mitochondrial network, thereby allowing mtDNAs to be released into the cytosol^25^. Cytosolic mtDNAs then activate the IFN-I response and induce the transcription of IFN-stimulated genes (ISGs)^25^. Notably, depletion of the YME1L protein boosted ISG expression even in BAX-/BAK-depleted cells. At the same time, treatment with VDAC inhibitor VBIT-4 impaired the induction of ISGs in YME1L-depleted cells, suggesting that mtDNAs were released from mitochondria mainly through VDAC1 pores^25^.

In contrast, multiple studies have shown that mtRNAs, in particular mt-dsRNAs, are released from the mitochondrial matrix and enter the cytosol via BAX/BAK macropores and MOMP. In cells deficient of polyribonucleotide nucleotidyltransferase 1 (PNPT1), an essential component of the mtRNA degradosome (mtEXO), the levels of mt-dsRNAs were increased and their cytosolic release was facilitated^11^. In particular, the release of mt-dsRNAs was nearly completely blocked by the downregulation of BAX and BAK^11^. In addition, stressors such as exogenous dsRNAs, mitochondrial dysfunction through the inhibition of ATP synthase, DNA damage, and oxidative stress can disrupt mitochondrial membrane potential to trigger the cytosolic release of mt-dsRNAs^11,26^. After release into the cytosol, these mt-dsRNAs are recognized by dsRNA-sensing PRRs such as MDA5, RIG-I, protein kinase R (PKR), and toll-like receptor 3 (TLR3) to trigger antiviral signaling^11,12,26,27^. Notably, a recent study showed that the unfolded protein response in the endoplasmic reticulum (ER) did not result in the release of mt-dsRNAs into the cytosol^28^, despite another study demonstrating that ER stress, exacerbated by obesity, led to mitochondrial damage in human adipocytes^29^.

### Innate immune activation by mtDNAs under various pathological conditions

Under stress conditions, mitochondrion-derived danger-associated molecular patterns (DAMPs), such as mtDNAs, succinate, N-formyl peptides, ROS, cardiolipin, and ATP, are released from mitochondria and activate several immune response pathways^30^. In particular, mtDNAs function as potent agonists of PRRs when present in the cytosol or in the extracellular environment^9^. Herein, we describe how cytosolic mtDNAs, released in response to various pathological stressors, trigger a cascade of innate immune responses, such as cGAS-stimulator of IFN genes (STING) expression, TLR9 pathway activation, and cytosolic inflammasome formation.

#### cGAS-STING PATHWAY

The structure and signaling mechanisms of cGAS-STING have been extensively reviewed in recent years^31,32^. Structurally, cGAS contains three dsDNA-binding sites and recognizes both self- and non-self-DNA in a sequence-independent manner^31^. cGAS monomers interact with mtDNAs longer than 45 bp and generate stable ladder-like networks of dimers^33^, leading to the activation of cGAS. Upon activation, cGAS converts GTP and ATP into 2′ 3′-cyclic GMP-AMP (cGAMP), which then binds to STING located on the ER and induces the conformational change of this ER protein. Then, STING is translocated to the Golgi apparatus and subsequently activates NF-κB and IRF3 pathways^33^. mtDNAs can also form immunostimulatory structures known as Z-DNAs, which are non-canonical structures with a high frequency of alternating purine–pyrimidine regions^34^. Mitochondrial genome instability promotes Z-DNA accumulation, and Z-DNA binding protein 1 (ZBP1) stabilizes the structure and recruits cGAS to induce IFN-I signaling^34^. The activation of the cGAS-STING pathway via cytosolic mtDNAs has also been linked to adaptive immunity by activating macrophages^35^. During bacterial clearance, neutrophil extracellular trap formation results in apoptosis and the extrusion of mtDNAs into the extracellular space^35^. Macrophages then engulf these extracellular mtDNAs to trigger the expression of IFN-I in a cGAS-dependent mechanism, resulting in abnormal macrophage activation^35^.

The cGAS-STING pathway and mtDNAs are involved in the pathogenesis of several conditions and cases of organ failure. High cGAS-STING activity and IFN-I response were observed in lung samples of SARS-CoV-2-infected patients with severe tissue damage^36^. Pharmacological inhibition of STING alleviated lung inflammation and improved disease prognosis in a mouse model of SARS-CoV-2^36^. The activation of the cGAS-STING pathway was also evident during liver inflammation, such as that observed in non-alcoholic fatty acid liver disease (NAFLD), hepatocellular carcinoma, cirrhosis, and viral hepatitis^37^. In the liver, the cGAS-STING pathway induces the progression of NAFLD to non-alcoholic steatohepatitis (NASH) in Kupffer cells (KCs)^38^. A study by Chen et al. showed that *Sam50* gene was essential for maintaining mitochondrial membrane integrity and mtDNA stability in hepatocytes^39^. Liver-specific knockout of *Sam50* in mice led to increased mitochondrial and cytosolic mtDNA levels, which resulted in the activation of the cGAS-STING pathway and subsequent liver inflammation^39^.

In cases of acute kidney injury (AKI), a nephrotoxic reagent induced mtDNA leakage into the cytosol and activated cGAS-STING signaling, ultimately resulting in renal tubular inflammation^40^. Indeed, STING deficiency ameliorated the kidney damage and inflammation induced by nephrotoxic cisplatin^40^. In addition, macrophages from rheumatoid arthritis (RA) patients expressed tumor necrosis factor (TNF), which inhibited PTEN-induced putative kinase 1 (PINK1)-mediated mitophagy and altered mitochondrial function to elevate cytosolic mtDNA levels^41^. mtDNA-induced cGAS-STING pathway activation has also been implicated in multiple neurodegenerative disorders, notably amyotrophic lateral sclerosis (ALS), a condition characterized by the cytosolic accumulation of TAR DNA-binding protein 43 (TDP-43)^42^. In neurons of ALS patients, TDP-43 invades mitochondria, which results in the release of mtDNAs via mPTPs and subsequent neuroinflammation^42^. Consistent with this mechanism, the level of cGAS signaling-related metabolites is significantly upregulated in the spinal cords of ALS patients^42^.

#### TLR9 PATHWAY

The cytosolic and extracellular mtDNAs can be sensed by TLR9, which is an immune cell (neutrophils, macrophages, and dendritic cells)-specific receptor located in the ER^43^. TLR9 directly senses hypomethylated CpG motifs in early endosomes^43^. As CpG motifs of endogenous mtDNAs are unmethylated, mtDNAs function as strong agonists for TLR9. When bound to mtDNAs, TLR9 recruits myeloid differentiation factor 88 (MyD88) and forms a signaling complex with interleukin-1 (IL-1) receptor-associated kinases (IRAKs) and tumor necrosis factor receptor-associated factor 6 (TRAF6) as well as with IRF7, transforming growth factor-β-activated kinase 1 (TAK1)-NF-κB inhibitor IκB (IκKBα) or TAK1-mitogen-activated protein kinase 1 (MAPK1) to promote proinflammatory gene expression via the activation of the transcription factor IRF7, NF-κB or activating protein-1 (AP-1)^44^. During SARS-CoV-2 infection, the mitochondrial network is impaired, and the released mtDNAs activated TLR9 signaling in endothelial cells, which exacerbates disease progression^45^. The release of mtDNAs also plays a critical role in the severity of SARS-CoV-2 infection and the initiation of sterile systemic inflammatory response syndrome by activating the TLR9/NF-κB pathway, resulting in the overproduction of proinflammatory cytokines^45,46^.

#### Cytosolic Inflammasomes

Finally, cytosolic mtDNAs contribute to the activation of inflammasomes, such as nucleotide-binding and oligomerization domain-like receptor family pyrin domain-containing 3 (NLRP3) and absent in melanoma 2 (AIM2), which facilitate the secretion of proinflammatory cytokines^9^. NLRP3 is a cytosolic PRR that is activated in response to a broad range of pathogen-derived and endogenous agents. In response to DAMPs, NLRP3 undergoes conformational changes to expose its central nucleotide-binding and oligomerization domain, promoting its oligomerization. AIM2 is another cytosolic receptor that recognizes dsDNAs and activates the inflammasome cascade. Extracellular dsDNAs released from necrotic cells are engulfed and recognized in cells by the AIM2 inflammasome, which drives pyroptosis and proteolytic maturation of the proinflammatory cytokines such as IL-18 and IL-1β^47^. Thus, cytosolic mtDNAs are ideal endogenous agonists that trigger NLRP3/AIM2 inflammasomes during necrosis or under cellular stress conditions. Once NLRP3/AIM2 inflammasomes are assembled, pro-caspase-1 is autocleaved, and the activated caspase drives the maturation and secretion of proinflammatory cytokines^48^. NLRP3/AIM2 inflammasome-activated caspase-1 also cleaves Gasdermin D (GSDMD), releasing the N-fragment of GSDMD, which in turn forms cell membrane pores to facilitate pyroptosis^49^.

**Fig. 2:**
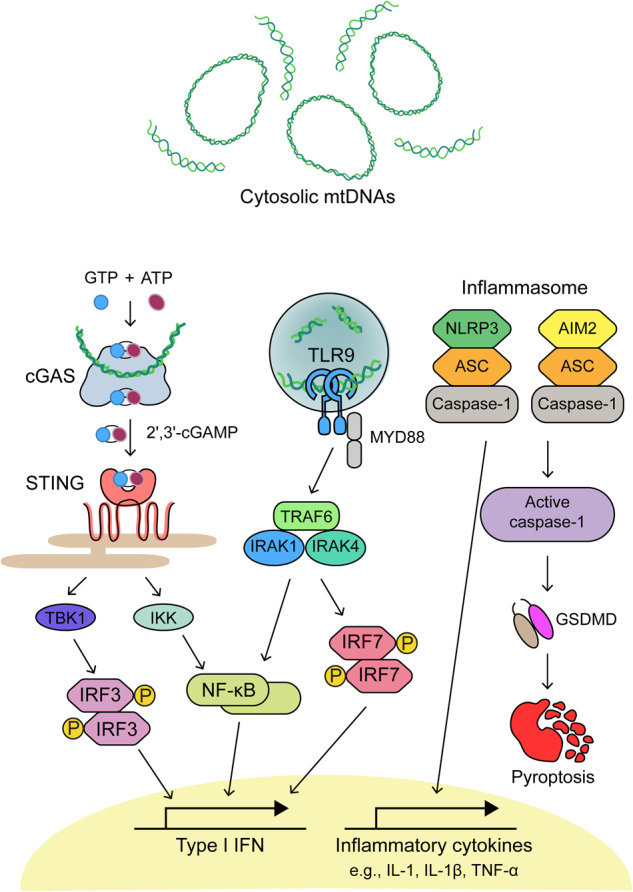
mtDNA-mediated immune signaling pathways. cGAS interacts with circular mtDNAs to form cGAMP, which then binds to STING on the ER. STING promotes the phosphorylation and dimerization of IRF3 through the actions of TBK1 and IKK to activate inflammatory gene expression. Upon mtDNA recognition, TLR9 in endosomal compartments activates NF-κB and IRF7 through the MyD88-dependent pathway and controls the expression of IFN-Is. Finally, mtDNAs activate cytosolic inflammasomes, leading to the maturation and secretion of inflammatory cytokines as well as the cleavage of caspase-1 to promote GSDMD-mediated pyroptosis.

The activation of cytosolic inflammasomes plays a critical role in regulating disease pathogenesis, such as acute myocardial infarction, systemic lupus erythematosus (SLE), inflammatory bowel disease such as Crohn’s disease, and bacterial infections^50^. Moreover, the increased production of IL-18 and IL-1β mediated by inflammasomes contributes to the onset and progression of RA, type II diabetes, and various neurodegenerative disorders, including Alzheimer’s disease (AD) and ALS^51^. The levels of AIM2 inflammasome-related proteins in the liver and those of IL-18 and IL-1β in serum were elevated in high-fat diet (HFD)-induced NAFLD model mice^52^. Moreover, mtDNA treatment promoted palmitate-induced activation of the AIM2 inflammasome and, subsequently, hepatocyte pyroptosis-exacerbated NAFLD^52^. In patients with type II diabetes, circulating cell-free mtDNAs induce AIM2 inflammasome-dependent caspase-1 activation and an increase in secreted IL-18 and IL-1β from macrophages^53^. Collectively, these studies suggest the close association of mtDNAs in human pathological conditions with inflammasome activation (Fig. 2).

### Innate immune response to mt-dsRNAs

There is accumulating evidence that mtRNAs, in particular mt-dsRNAs, are important DAMPs that can activate the innate immune response and inflammatory pathways. Each cell contains thousands of copies of circular mtDNA genomes, which generate long dsRNAs via bidirectional transcription. The two complementary strands of the circular mitochondrial genome, the heavy (H) and the light (L) strands, are transcribed when mitochondrial transcription factor A (TFAM) recruits mitochondrial RNA polymerase (POLRMT) to promoters (denoted as HSP and LSP) via the POLRMT N-terminal extension^54^. The resulting two complementary strands of mtRNAs can bind to each other and form mt-dsRNAs that can be as long as the entire mitochondrial genome^10^.

#### mt-dsRNA recognition by dsRNA-sensing PRRs

Once released into the cytosol, mt-dsRNAs are detected by RLRs, such as MDA5 and RIG-I, which initiate the IFN-I response. The domain structures in MDA5 and RIG-I are similar, although MDA5 preferentially binds long dsRNAs, which activate it^55^. MDA5 interacts with dsRNAs through the C-terminal domain by binding to the RNA phosphate backbone and 2′-hydroxyl groups, resulting in sequence-independent RNA recognition^55^. The extended duplex structure of mt-dsRNAs serves as a platform for MDA5 oligomerization, which is required for MDA5 activation. As another member of the RLR family, RIG-I interacts with dsRNAs derived from mitochondria as well. However, RIG-I recognizes short blunt-ended dsRNAs bearing 5′-triphosphate or can also bind 5′-diphosphate dsRNAs, but with lower affinity than dsRNAs with 5′-triphosphate^55^. Transcription of short mtDNAs due to DNA double-stranded break results in the transcription run-off and generation of short dsRNAs carrying a 5′-end triphosphate, ideal substrates for RIG-I^55^. Thus, the mitochondrial genome can generate both long and short mt-dsRNAs that can activate MDA5 and RIG-I, respectively. Once activated, the two proteins transmit signals through MAVS to initiate a downstream innate immune response^55^.

PKR is another key cytosolic dsRNA sensor that interacts with mt-dsRNAs. PKR binding to dsRNAs is mediated through two dsRNA-binding domains, which results in the dimerization and the subsequent autophosphorylation of key residues, including Thr446 and Thr451^56^. Activated PKR binds to a conserved site in the alpha subunit of eukaryotic initiation factor 2 (eIF2α) and induces eIF2α phosphorylation at Ser51, blocking translation at the initiation step^56^. Moreover, PKR activation triggers the expression of inflammatory cytokine and IFN-I mediated by the IKK/NF-κB and TAK1/AP-1 pathways. Recently, many studies have revealed the roles of mt-dsRNAs as key activators of PKR in numerous human pathologies. During osteoarthritis development, mitochondrial dysfunction results in the cytosolic accumulation of mt-dsRNAs, which activate PKR and initiate apoptosis in chondrocytes^26^. Notably, blocking mtRNA transcription using a small chemical inhibitor of POLRMT, 2-C′-methyladenosine (2-CM), decreased the level of mtRNAs and attenuated PKR phosphorylation under osteoarthritis-mimicking stress conditions^26^. Similarly, PKR activation mediated by mt-dsRNAs has been reported in the context of autoimmune Sjögren’s disease (SjD)^57^. Specifically, polyinosinic-polycytidylic acid (poly I:C), a synthetic analog of viral dsRNAs, was used to stimulate IL-7 production and early inflammatory responses in salivary gland acinar cells to establish a model of SjD-like sialadenitis^58^. Interestingly, poly I:C stimulation led to the elevation of total and cytosolic mt-dsRNA levels, subsequent PKR phosphorylation, and ISG induction, although the molecular mechanism that drives the elevation of mt-dsRNAs remains to be investigated^57^. In this context, cytosolic mt-dsRNAs acted as positive feedback factors to augment the antiviral signaling initiated by poly I:C. Finally, the release of mt-dsRNAs and concomitant upregulation of innate immune signaling were observed in neurodegenerative disorder Huntington’s disease (HD) patients, where the mt-dsRNAs directly bound, activated PKR, and induced inflammatory response as well as programmed cell death in neurons^59^_._

In the endosomal pathway, TLR3 recognizes mt-dsRNAs present in the extracellular space as both free-form and in exosomes. Studies showed that extracellular mt-dsRNAs undergo endocytosis and are transported into the endosomal lumen where they function as ligands of TLR3^26,27^. Once TLR3 senses dsRNAs, the toll-IL-1 receptor (TIR) domain undergoes a conformational change and its tyrosine residues are phosphorylated, resulting in the formation of dually phosphorylated TLR3^60^. Phosphorylated TLR3 then induces the oligomerization of TIR-domain-containing adapter-inducing IFN-β (TRIF), which subsequently activates IRF3 and NF-κB to produce proinflammatory cytokines and IFN-Is^60^. In an alcoholic liver disease (ALD) mouse model, extracellular mt-dsRNAs in exosomes were implicated in the increased expression of IL-1β and interleukins, especially IL-17A, in KCs^27^. In this context, the alcohol-associated stress in hepatocytes led to accumulated mt-dsRNAs in the cytosol and in the extracellular space due to a significant decrease in the expression of PNPT1. Another study showed that the overexpression of heat shock protein 60 (HSP60) alleviated HFD-elicited mtDNA and mt-dsRNA release into the extracellular space, thereby preventing the recognition of mt-NAs by TLR3 and MDA5 in the liver^61^. Moreover, mitochondrial dysfunction during osteoarthritis development resulted in the release of exosomal and cell-free mt-dsRNAs, which activated TLR3 and induced the expression of downstream factors, such as *IRF3*, *TRAF3*, and *LY6E* mRNAs in neighboring chondrocytes^26^. The implication of these cell line data was further supported by an analysis of synovial fluids from osteoarthritis patients, which showed elevated levels of mt-dsRNAs compared to those of mRNAs or noncoding RNAs (ncRNAs)^26^. Overall, the activation of PRRs by mt-dsRNAs may drive the development of several human diseases, including ALD^27^, HD^59^, and osteoarthritis^26^.

#### Autoantibodies associated with mtRNAs

**Fig. 3:**
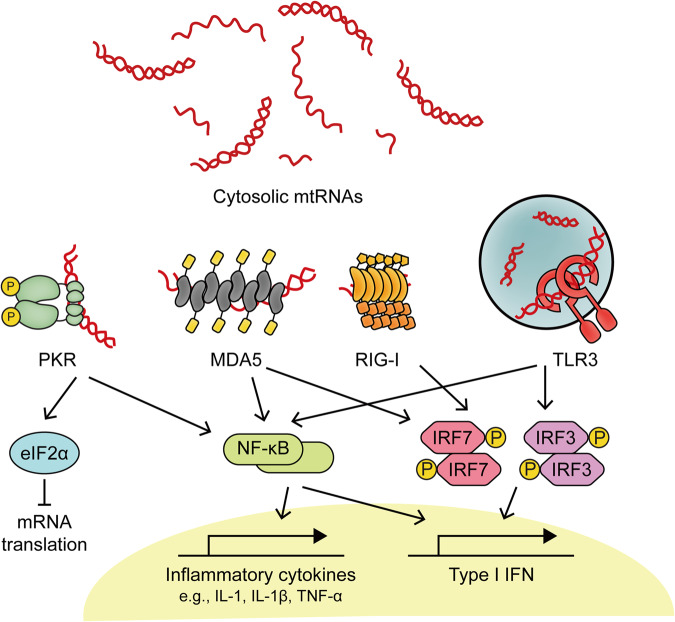
mt-dsRNA-mediated immune signaling pathways. Cytosolic mt-dsRNAs can activate PKR, MDA5, and RIG-I to initiate apoptotic programs as well as IFN-I and inflammatory responses. In addition, extracellular mt-dsRNAs are recognized by TLR3, resulting in the production of IFN-Is and proinflammatory cytokines.

Interestingly, mtRNAs are also recognized by autoantibodies characteristically generated in the context of autoimmune disorders. In SLE patients, both immunoglobulin G (IgG) and IgM subclass autoantibodies targeting mtRNAs have been detected^62^. These findings suggest that mtRNAs may be mitochondrial neoantigens and support the idea that mitochondria are important sources of circulating autoantigens in patients with SLE^62^. In addition, mt-dsRNAs are closely associated with the downstream response to an increase in the number of autoantibodies in SjD patients^57^. Under physiological conditions, acetylcholine (Ach), the endogenous ligand for muscarinic acetylcholine receptor subtype 3 (M3R), suppresses ISG induction partially by preventing the cytosolic release of mt-NAs^63^. However, SjD patients show an elevated level of anti-M3R autoantibodies, which counter the protective effect of Ach, aggravating the antiviral responses^57^. Collectively, studies show that mt-dsRNAs are potent cytosolic and extracellular activators of dsRNA sensors and are associated with a range of human diseases that are accompanied by inflammation and cell death (Fig. 3).

### Regulation of mt-NA-mediated immune activation

The efflux of mt-NAs and their immunostimulatory interactions with molecular sensors provoke critical activation of innate immune signaling pathways. To counter the potentially harmful effects, including apoptosis, caused by mitochondrial failure, cells have adapted countermeasures to prevent and resolve stress arising from the aberrant accumulation of mt-NAs. mtDNA transcription and replication give rise to mitochondrial R-loop and G-quadruplexes (G4) structures, which show immunostimulatory effects. R-loops are nucleic acid structures composed of an RNA–DNA hybrid and a displaced ssDNA. Silva et al. reported that the persistent and inadvertent formation of R-loops blocked replication and transcription machinery activity, leading to genome instability and, ultimately, the induction of human diseases such as neurodegenerative disorders and cancer^64^. Moreover, the mtEXO, formed by human suppressor of var1,3-like 1 (hSUV3) and PNPT1, is a guardian of mitochondrial genome integrity that counteracts the excessive accumulation of mitochondrial DNA–RNA hybrids^64^. In this context, an mtEXO binds and processes new transcripts to deter DNA–RNA hybridization and thus preserves individual DNA molecules. Alternatively, an mtEXO may play a more direct role where hSUV3 helicase unwinds the hybridized RNA moiety, which allows its immediate degradation by PNPT1^64^. When DNA–RNA hybrids are not degraded, long stretches of cytosolic DNA–RNA hybrids are bound to cGAS, which triggers cGAS-STING-dependent expression of antiviral genes^65^.

In addition to R-loops, some mt-NA sequences rich in guanines adopt a non-canonical G4 conformation^66^. The guanine-rich heavy strand is a potential hotspot for G4 formation, which disrupts mitochondrial replication and transcription machinery actions^66^. Specifically, LSP transcription can be arrested by a hybrid DNA–RNA G4 structure formed at conserved block sequences^66^. Moreover, G4s are linked to origins of the mitochondrial genome instability and mtDNA deletion associated with human diseases, such as renal cell carcinomas^67^. Therefore, mitochondrial G4 structures can potentially be utilized as predictive markers for cancer associated with abnormal mtDNA metabolism^67^. Considering their high copy number and gene density, mtDNAs have the potential to amplify the effects of G4 dysregulation, resulting in compromised mitochondrial respiratory function and cell viability^66^. Hence, it is critical to identify and predict potential mitochondrial G4s and the proteins with which they interact to prevent defects in the mitochondrial genome.

The abundance of mt-dsRNAs is also regulated to prevent ectopic activation of immune responses. One of the regulatory proteins involved is RNA exonuclease 2 (REXO2), a 3′-to-5′ exonuclease that degrades small RNAs generated during mtRNA processing in the mitochondrial matrix^68^. REXO2 prevents the aberrant accumulation of short mt-dsRNAs that can induce an IFN response or the formation of R-loops that interfere with mtDNA maintenance^68^. Mitochondrial poly(A) polymerase (MTPAP) adds short poly(A) tails to the 3′ ends of most H- and L-strand RNAs to stabilize them^69^. Similarly, leucine-rich pentatricopeptide repeat motif-containing protein (LRPPRC) interacts with steroid receptor RNA activator-stem loop-interacting RNA-binding protein (SLIRP) via a PPR–RNA recognition motif interface^70^ to form a dimer that protects mtRNAs from mtEXO by providing a physical barrier^71^. The loss of LRPPRC also results in transcript-specific alterations in the steady-state levels of mtRNAs and their poly(A) tail length^72^, thereby suggesting that the LRPPRC/SLIRP complex acts as an RNA molecular chaperon to promote RNA polyadenylation and thus protects RNA from degradation^72^.

Another important layer of posttranscriptional regulation of mtRNAs is provided by mitochondrial RNA granules (MRGs)^73^. MRGs are fluid-like condensates involved in RNA processing and degradation mediated by the mtEXO^74^. Specifically, hSUV3 unwinds RNA substrates, and the phosphate-dependent 3′-to-5′ exonuclease PNPT1 preferentially degrades the mtRNA strand transcribed from the L-strand^11^. Indeed, the downregulation of PNPT1 and/or hSUV3 results in the stabilization of L-strand mtRNAs and accumulation of mt-dsRNAs^11,74^. Recently, increasing attention has been focused on the protective role of PNPT1 in the mt-dsRNA-induced IFN response, emphasizing its importance in human health. Pathogenic mutations in both *PNPT1* and *hSUV3* genes led to the mt-dsRNA accumulation, which has been implicated in neurodegenerative syndromes, including Aicardi–Goutières syndrome^75,76^. Furthermore, an isoform of an mtRNA-binding protein, G-rich sequence factor 1 (GRSF1), regulates nascent RNA processing in MRGs^77^. Foci in the mitochondrial matrix contain GRSF1, nascent mtRNA, and RNase P, which is an enzyme responsible for classical and tRNA-less RNA precursor processing^78^. Defects in GRSF1-mediated mtRNA processing have been associated with several mitochondrial disorders in hepatocytes and OXPHOS dysfunction in skeletal muscles and bone marrows^77^.

### mt-NAs as potential therapeutic targets

Due to their strong immunogenicity, mt-NAs are emerging as potential therapeutic targets to alleviate inflammatory responses. For example, inhibiting VDAC1 oligomerization by VBIT-4 attenuated immune activation and lupus-like symptoms by preventing the cytosolic release of mtDNAs^17^. In addition, factors mediating the downstream response to cytosolic mt-NAs can be targeted. One study showed that doxorubicin, a DNA-damaging chemotherapeutic agent, induced mtDNA instability and Z-DNA accumulation in cardiomyocytes, which triggered a robust cardiac IFN-I response that was dependent on ZBP1, STING, and IFNAR^34^. By targeting ZBP1, the cardiotoxicity induced by doxorubicin was reduced and heart function was improved, indicating that ZBP1 is a potential therapeutic target to prevent chemotherapy-related heart failure^34^.

Leveraging mitophagy to remove damaged mitochondria and their immunogenic debris is an alternative strategy for modulating mt-NA-mediated immune activation. The inhibition of mitophagy by downregulating PINK1 triggered the accumulation of mtDNAs in the cytosol, exacerbating RA-related symptoms^41^. By exploiting this effect, Xie et al. proposed targeting PINK1 as a strategy to enhance therapeutic responses to chemoimmunotherapy^79^. Similarly, autophagy can remove cytosolic mt-dsRNAs to prevent triggering a downstream immune response. Indeed, autophagy inducers, such as torin-1 and metformin, reduced the cytosolic level of mt-dsRNAs that had accumulated in response to mitochondrial stress in chondrocytes^26^. The decrease in mt-dsRNA levels subsequently attenuated the IFN-I response and rescued cell death. More importantly, this rescuing effect of autophagy inducers was diminished in mtRNA-deficient cells, suggesting that the effects were mediated partly by alterations to mtRNA levels^26^.

The expression of mtRNAs can be directly modulated. A recent study revealed a significant decrease in mt-dsRNA production and subsequent immunosuppression in breast cancer cells under hypoxic conditions^80^. This outcome suggests a novel mechanism of immunosuppression that can be utilized by hypoxia-inducing therapies^80^. In cell models of osteoarthritis and SjD, the attenuation of IFN signatures has been observed when the expression of mtRNAs was suppressed by treating the cells with 2-CM, an inhibitor of POLRMT^26,57^. In addition to 2-CM, a first-in-class specific inhibitor of mitochondrial transcription (IMT1B) has been developed, by Bonekamp et al.^81^, and it may be an alternative drug used for downregulating mt-dsRNA expression. In fact, IMT1B showed potent antitumor properties, and IMT1B treatment led to progressive loss of mtDNA levels and increased cell death^82^. Targeting POLRMT via IMT1B also suppressed endometrial carcinoma growth by impairing mitochondrial function, leading to mtDNA transcription inhibition, decreased mitochondrial membrane potential, ROS production, oxidative stress, and ATP loss^83^. Overall, modulating the expression of mtRNAs is a promising therapeutic strategy for not only attenuating symptoms and treating inflammatory diseases but also for enhancing the efficacy of chemoimmunotherapy.

Extracellular vesicles (EVs) enriched with mitochondrial contents (mitoEVs) are emerging as therapeutic materials. These vesicles deliver mitochondrial components that trigger the release mitochondrial DAMPs and alter cell signaling pathways in recipient cells under pathological conditions, such as inflammation, cancers, and lung diseases^84^. For instance, acute myeloid leukemia cells showed enriched levels of mitochondria in microvesicles (MVs) during differentiation, and inhibiting MV formation prevented myeloid differentiation^85^. In addition, mouse melanoma cells released mtDNA-rich EVs to induce cytokine production in macrophages, which suppressed the immune responses in the tumor microenvironment triggered by cytotoxic T cells^86^. On the other hand, mitoEVs from healthy cells are promising therapeutics for lung injuries because of their metabolic and/or immune regulatory effects^84^. For example, mitoEVs derived from the mesenchymal stem cells delivered healthy mitochondria to improve the oxygen consumption rate as well as the mtDNA and ATP levels in alveolar macrophages^87^. Considering that EV-mediated delivery of mt-NAs can be achieved in cells under either a physiological or pathological state, mitoEVs have promising diagnostic and therapeutic potential.

### Physiological roles of mt-NAs

Due to their strong association with pathologies, it is unclear why mitochondria carry unmethylated circular DNAs and generate long dsRNAs that are detrimental to cells when released to the cytosol, as described above. Hence, discerning the potential physiological roles of mt-NAs remains as a challenge in the field. Recent studies have revealed strategies that cells employ to evade mt-NA-mediated immune activation and utilize cytosolic mt-NAs as beneficial signaling molecules. In particular, an increasing number of studies have reported on the role of mt-NAs during adipogenesis. Herein, using adipogenesis and beige adipocytes as case studies, we describe how mt-NAs can be used as upstream cues to trigger non-IFN responses.

Adipose tissue plays a crucial role in metabolic regulation and homeostasis by controlling energy storage and expenditure. White adipose tissue (WAT) and brown adipose tissue are the two main types of fat tissue, with the former functioning as a reservoir of energy in a single large lipid droplets and the latter dissipating energy as heat through nonshivering thermogenesis mediated with highly dense mitochondria^88^. Additionally, a thermogenic population of adipocytes, known as beige adipose tissue, is also found interspersed among subcutaneous WATs (scWATs). During adipose tissue remodeling of browning, scWAT acquires brown fat-like features, such as an elevated level of the thermogenic mitochondrial protein uncoupling protein 1 (UCP1) and an increased number of mitochondria^89^. Even in adipocytes, dysregulation or considerable abundance of mitochondria is associated with the release of mt-NAs into the cytosol^13^. Impaired mitochondrial respiration and enhanced ROS production in adipose tissue have ben related to the cytosolic release of mtDNAs, which increase the possibility of STING-dependent inflammation and insulin resistance under conditions of obesity^90^. Indeed, the injection of mtDNAs or mtRNAs into the cytosol of adipocytes triggered an increase in Ifn-β expression in adult mice^13^. In turn, IFN immune responses damaged mitochondrial capacity for fat oxidation and thermogenesis, leading to inflammation and insulin resistance^13^.

During beige adipocyte differentiation, a developmental program that mitigates the potential risk of mtRNA efflux is activated because of an increase in the number of mitochondria^13^. Interestingly, despite carrying more mitochondria, adipocytes of young mice do not show Ifn responses after stimulation by mtRNAs due to the suppression of Irf7 production by vitamin D receptor (Vdr) signaling^13^. In adult mouse inguinal adipocytes, the Irf7-associated gene network, including ISGs such as *Aim2, Ddx41, Zbp1,* and *Ifi205g* genes, was highly expressed, promoting the initiation of the Ifn response to the increase in cytosolic mt-NA levels^13^. Consistent with these results observed in mice, the results observed in the adipocytes of lean children (1-4 years) revealed reduced expression of IFN-β in response to cytosolic mtRNAs compared to that of adipocytes of adolescents (16-17 years)^13^. In beige adipocytes of young mice, mtRNAs contributed to mitogenesis and mitochondrial thermogenesis under specific conditions but not to an Ifn response^13^. Cytosolic mtRNAs upregulated the expression of Ucp1, a key player in thermogenic mitochondrial function, and other beige adipocyte marker genes, namely, *Ppargc1a, Cidea*, and *Dio2*^13^. Therefore, they increased the thermogenic activity of mitochondria. The absence of dsRNA sensors impeded the induction of thermogenic gene expression and ultimately resulted in the loss of beige adipocytes in young mice, indicating that the effects of cytosolic mtRNAs were facilitated through the dsRNA structure^13^. This study suggests the involvement of the *Il-6* gene in the mtRNA-mediated control of adipocyte remodeling, as this gene enhances thermogenic adipocyte development^13^. The dsRNA sensors activated by mtRNAs increase Il-6 expression, and the inhibition of Il-6 signaling compromises the effect of mtRNAs on the beige adipocyte gene expression^13^. The molecular mechanisms underlying mtRNA-triggered signaling, originating in mitochondria and controlling transcription in the nucleus, remain to be characterized.

**Fig. 4:**
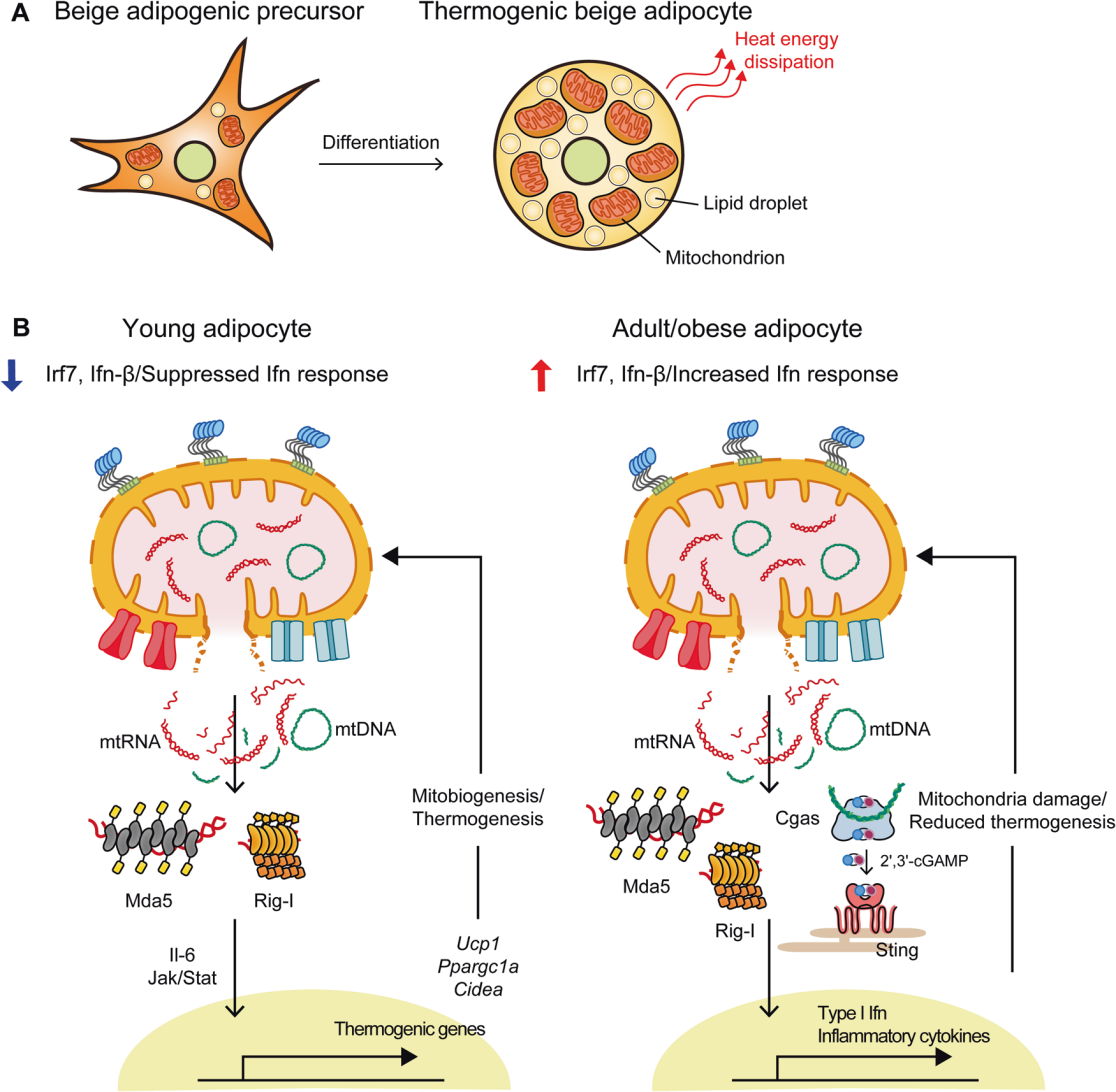
mt-NAs-mediated regulation of thermogenic adipocytes. **A** Mitochondria and small lipid vacuoles accumulate as adipogenic precursors differentiate into mature beige adipocytes. Mature beige adipocytes dissipate heat through nonshivering thermogenesis, which increases energy expenditure. **B** mtRNA-mediated stimulation of thermogenesis in young adipocytes with a suppressed Ifn response. Cytosolic mtRNAs recognized by dsRNA sensors activate thermogenic adipocyte differentiation through Il-6-Jak/Stat signaling in young mice and humans. In older or obese mice/humans, cytosolic mt-NAs induce an Ifn response and abrogate the expression of genes related to thermogenesis.

The impact of mtDNAs on thermogenic gene expression in adipocytes was revealed by Bai et al., who examined the effect of fat-specific knockout of the disulfide bond A oxidoreductase-like gene (*DsbA-L*), which encodes a chaperone-like mitochondrial protein^91^. *DsbA-L* knockout promoted the cytosolic release of mtDNAs, triggering the activation of the cGAS-STING pathway. As a result, the expression of *Ucp1, Ppargc1a, Cebpb*, and *Prdm16*, genes associated with the thermogenic function of beige and brown adipocytes, was suppressed, leading to decreased thermogenesis and increased adiposity^91^. The cGAS-STING pathway mediated the effects of *DsbA-L* knockout on thermogenic gene expression and metabolic phenotype acquisition by inhibiting protein kinase A (PKA) signaling through the downregulation of phosphodiesterase (PDE)-dependent adenosine 3′,5′-cyclic monophosphate (cAMP)^91^. Additionally, Aim2, a cytosolic inflammasome, has been implicated in adipogenesis through the Ifn-inducible gene *Ifi202b*^92^. These findings suggest that mtDNA modulates adipogenic and thermogenic programs in conjunction with immune response regulation (Fig. 4).

A high mtDNA copy number is associated with the potential physiological functions of mt-NAs. A decrease in the mtDNA copy number in the peripheral blood has been associated with chronic kidney diseases, suggesting the involvement of mtDNAs in renal function^93^. In addition, multiple mtDNA deletion mutations accumulate with age in tissues with high respiratory rates, such as the brain. A study by Nicholas et al. reported that the decreased expression of transcription factor B2 (TFB2M) led to increased risk of diabetes by disrupting the transcription of mtDNAs and reducing the mtDNA content^94^. Although the exact mechanism and the key factors regulating mt-NA levels in specific cellular and physiological contexts remain to be investigated, recent findings have implicated mt-NAs in many processes related to cell physiology.

## Conclusion

This review summarizes the critical role of mt-NAs as endogenous ligands that activate various innate immune signaling pathways. In addition, it describes the potential physiological function of mt-NAs, especially during beige adipocyte differentiation. When released to the cytosol via MOMP, mtDNAs initiate cell signaling pathways such as the cGAS-STING and TLR9 pathways and induce inflammasome assembly, leading to the activation of transcription factors such as IRF3 and NF-κB and the production of IFN-Is and inflammatory cytokines. Furthermore, mt-dsRNAs, generated via the bidirectional transcription of the circular mitochondrial genome, are recognized by dsRNA sensors, such as MDA5, RIG-I, PKR, and TLR3, which induce the expression of proinflammatory cytokines, including IFN-Is, and mediate cell death via global suppression of translation. The recognition of mt-NAs by PRRs and the human diseases associated with mt-NAs are summarized in Table 1, along with information about the potential therapeutic benefits of targeting mt-NAs and their downstream signaling in Table 2.

**Table 1:** Summary of mt-NAs, their interacting receptors, and the associated human diseases.

mt-NA	Interacting receptors	Associated diseases
mtDNA	cGAS	SARS-CoV-2^36^ Nonalcoholic steatohepatitis^38^ Acute kidney injury^40^ Rheumatoid arthritis^41^ Amyotrophic lateral sclerosis^42^
	TLR9	SARS-CoV-2 infection^45^ Systemic inflammatory response syndrome^46^
	NLRP3	Acute myocardial infarction^50^ Systemic lupus erythematosus^50^ Rheumatoid arthritis^51^ Type II diabetes^51^ Parkinson’s disease, Alzheimer’s disease, and multiple sclerosis^51^
	AIM2	Nonalcoholic fatty acid liver disease and hepatocyte pyroptosis^52^ Type II diabetes^53^
mtRNA	RLRs	Aberrant immune responses^11^
	TLR3	Alcoholic liver disease^27,61^ Osteoarthritis^26^
	PKR	Osteoarthritis^26^ Sjögren’s disease^57^ Huntington’s disease^59^

**Table 2:** Summary of potential therapeutic effects expected by targeting mt-NAs.

Associated diseases	mt-NA-mediated therapeutic effects	
	Target	Potential therapeutic effects
SARS-CoV-2	STING^36^	STING inhibition reduces SARS-CoV-2-induced inflammation in mice.
	TLR9^45^	Targeting TLR9 rescues compromised endothelial cell function due to reduced expression of nitric oxide synthase.
Non-alcoholic steatohepatitis	STING^38^	STING functions as an mtDNA sensor in KCs of the liver under lipid overload to induce NF-κB-dependent inflammation in the context of NASH.
Acute kidney injury	cGAS-STING^40^	Reduced activation of the cGAS-STING pathway prevents cytosolic mtDNA-mediated inflammation in cisplatin-induced AKI.
Rheumatoid arthritis	cGAS-STING^41^	cGAS deficiency ameliorates arthritic symptoms and reduces ISG induction.
	NLRP3 inflammasome^51^	Targeting NLRP3 or downstream caspases suppresses IL-1 production in the RA context.
Amyotrophic lateral sclerosis	cGAS-STING^42^	cGAS and STING inhibitors prevent TDP-43-induced inflammation and improve the symptoms of neuronal decline in patients with diseases involving TDP-43 proteinopathy.
Liver disease	TLR3^27^	Targeting mt-dsRNA-mediated TLR3 activation prevents the recruitment of γδ T cells and expression of IL‐17A in the early stage of ALD.
	HSP60^61^	Overexpression of HSP60 significantly reduces weight gain and fat accumulation in adipose tissue as well as mt-dsRNA-mediated inflammation in chronic kHFD.
Osteoarthritis	POLRMT^26^	Reduced expression of mt-dsRNAs by 2-CM treatment rescues the activation of PKR and eIF2α and reduces ISG induction.
Sjögren’s disease	POLRMT^57^	Downregulation of mtRNAs reduces the phosphorylation of PKR and attenuates ISG induction, which rescues the viability of salivary gland acinar cells.

mt-dsRNAs, in particular, are emerging as underlying immune activators that drive the pathogenesis of a wide range of human diseases, which opens up new insights and leading to questions that need to be answered. A recent report by Kim et al. described three complementary approaches to analyze the expression of mt-dsRNAs in multiple cell types^95^. Technical advancements in mt-dsRNA analysis will allow researchers to carry out the additional mechanistic and biochemical studies required to elucidate the formation of mt-dsRNAs, the posttranscriptional regulation of mt-dsRNAs, and the precise molecular structures that are recognized by PRRs. In addition to that directed to mt-dsRNAs, research is needed on the mitochondrial genome, which encodes long ncRNAs, small ncRNAs, and even circular RNAs^96^. Notably, a conserved 7S ncRNA transcribed from LSP inhibits mitochondrial transcription by functioning as a scaffold for POLRMT dimerization^97^. Moreover, other mitochondrial ncRNAs contribute to the regulation of mitochondrial gene expression, metabolism, and homeostasis^98^. Yet, the biological function of these mitochondrial ncRNAs remains to be investigated.

Recent studies highlighted the function of mt-NAs beyond innate immunity. Interestingly, mt-NAs serve as key mediators of the signaling crosstalk between mitochondria and the nucleus. The cellular signaling initiated by chronic mtDNA stress conditions triggers the nuclear DNA repair system and protects the nuclear genome^99^. The efflux of mtRNAs also stimulates the transcription of nuclear-encoded genes related to mitobiogenesis and thermogenesis in early adipocyte development^13^. Moreover, mtRNA modifications can alter cellular metabolic pathways and cell homeostasis, which depends on OXPHOS stimulation and oxidative stress neutralization^100^. Indeed, dysregulation of effectors of RNA modifications leads to metabolic alterations that are similar to those related to obesity phenotypes^100^. Additionally, mitochondrial tRNA modification accelerates the metastasis of head and neck cancer by functions as a metabolic switch^101^. In summary, investigating the biogenesis of mt-NAs, the posttranscriptional modifications that regulate mt-NA functions, and their downstream effects on cell signaling will allow us to further elucidate the important roles of mt-NAs under both physiological and pathological conditions.
